# Protective natural autoantibodies and lupus pathogenesis

**DOI:** 10.1186/ar4645

**Published:** 2014-09-18

**Authors:** Gregg J Silverman

**Affiliations:** 1 NYU School of Medicine, New York, NY, USA

## Background

Antibodies produced by B lymphocytes provide key functions that help protect against pathogens while recent studies show there can also be roles in maintaining homeostasis. Substantial levels of spontaneously arising IgM antibodies recognize cross-reactive oxidation-associated epitopes, such as phosphorylcholine (PC) on apoptotic cells. One class of these natural antibodies can enhance efficiency of apoptotic clearance by innate immune cells, and also suppress proinflammatory responses. *In vivo *administration can block the development of the inflammatory autoimmune disease and enhance survival of lupus-prone mice. These inhibitory responses are linked to anti-inflammatory signaling with nuclear localization of MAPK phosphatase-1, a factor known to also mediate glucocorticoid suppression of immune responses.

## Methods

To investigate the relevance to clinical SLE disease we analyzed the relationships between NAb levels with lupus disease activity, tissue injury and cardiovascular (CV) effects in two independent lupus cohorts.

## Results

In 120 SLE patients from the Hopkins cohort, levels of IgM anti-PC were significantly higher in patients with low disease activity and with less organ damage by SELENA SLEDAI, as well as by the physician's evaluation and the SLICC damage score. Importantly, IgM anti-PC levels were also significantly higher in patients without histories of clinical CV events (that is, MI, angina or stroke). In 105 SLE patients from the NYU cohort, we found that subclinical CV disease, as detected by carotid ultrasound, correlated with lower levels of IgM anti-PC (*P *= 0.004), and also lower ratios of IgM anti-PC/total IgM, compared with patients without plaque (*P *= 0.02). The IgM anti-PC/total IgM association remained significant after adjusting for age, cholesterol and hypertension. Adiponectin and sE-selectin were significantly elevated in patients with plaque, and statistical models showed that combining adiponectin, sE-selectin and IgM anti-PC/total IgM was better for predicting plaque than either test alone.

## Conclusions

Our clinical surveys have contributed to emerging evidence that regulatory anti-PC antibodies may oppose the influence of pathogenic lupus autoantibody ICs. Our data suggest that levels of anti-PC IgM antibodies may serve as a surrogate biomarker for vascular damage associated with subclinical atherosclerosis, and could have clinical applicability for monitoring and predicting relative risk in SLE patients.

